# Changes and protections of urban habitat quality in Shanghai of China

**DOI:** 10.1038/s41598-023-32247-7

**Published:** 2023-07-06

**Authors:** Zi-Xia Xie, Biao Zhang, Yun-Ting Shi, Xiu-Yu Zhang, Zi-Xin Sun

**Affiliations:** 1 Guangdong Provincial Academy of Environmental Science, Guangzhou, 510045 People’s Republic of China; 2 Institute of Geographical Sciences and Natural Resources Research, Chinese Academy of Science, Beijing, 100101 People’s Republic of China; 3 University of Chinese Academy of Sciences, Beijing, 100049 People’s Republic of China

**Keywords:** Urban ecology, Biodiversity

## Abstract

Habitat quality has been widely used as an important indicator in the evaluation of regional ecological security and ecosystem services. Previous studies have focused on the influences of urbanization on habitat quality, but the protection measures about how to respond to the dynamic changes of habitat quality patterns are still unclear. This study investigated the habitat quality in the metropolitan region of China (Shanghai) by using InVEST model, and analyzed its dynamic changes from 2000 to 2017 for the sake of providing different protection objects and measures for Shanghai. The results showed that the habitat quality index (HQI) in 2017 was 0.42, and the accumulated area percentages of less than 0.4 in HQI reached 46%, whereas the habitat quality in Chongming district was the highest. The HQI and habitat protected index (HPI) showed an obvious decline tendency from suburban area to downtown area. The HQI in Shanghai gradually declined from 0.56 in 2000 to 0.42 in 2017, and the deterioration area in habitat quality nearly covered 33% between 2000 and 2017. Additionally, the area proportion of the median habitat quality (0.4 < HQI ≤ 0.6) drastically dropped, but the areas of the low (HQI ≤ 0.2) and the high (HQI > 0.8) in habitat simultaneously expanded. Therefore, the valuable habitat in the western and southern coastal wetlands, Dianshan lake and Chongming district in Shanghai should be strictly protected, which covered 30% of the metropolitan area in Shanghai, and about 17% of the region located in the inner coastal zones and northern of Chongming Island was in urgent need of habitat restoration. Our results provide vital references for the maintenance and sustainable management of urban habitats in the metropolitan region.

## Introduction

Natural habitat is not only an important part of biodiversity, but also an essential basis for maintaining the stability and resilience of ecosystems^1^. However, rapid urban expansion is threatening the natural habitat in metropolitan regions^2,3^. For example, Mcdonald et al.^4^ found that relentless urban growth has increased threats to the biodiversity of native species, and the distance between protected areas and cities was predicted to shrink dramatically in Eastern Asia. The dramatic urban expansion in the Beijing–Tianjin–Hebei region has led to the decline of habitat quality and the fragmentation of habitat patches^5^, and a significant decline in habitat quality also occurred in Hangzhou and Changsha due to the changes of the landscape patterns caused by urban sprawl^6,7^.

Habitat quality determines the provision ability of the natural environment for suitable survival of individuals and sustainable development of populations, and often used as an important indicator of biodiversity^8^. Recently, the dynamic monitoring and assessment on habitat quality has been paid more attention^9–11^, and mainly through comprehensive index system at river or bay scale^12,13^, field survey^14^, or simulation models at regional scale^15^. However, the field survey method is often appropriate for single habitats in specific areas^12,13^, whereas difficult to be applied in long-term and large-scale research. The rapid development of remote sensing and geographic information techniques facilitated the integration of land use types and threat sources into simulation models of habitat quality, such as *ARIES*, *MIMES*, and *InVEST*. InVEST model assesses habitat quality by using data of land use types, habitat suitability, habitat sensitivity, and threat intensity of disturbance factors of each ecosystem type. It can be helpful in study areas where the species distribution data are poor or mixed habitat types co-exist. In addition, it can provide more detailed measures of biodiversity status and accurately evaluate spatiotemporal changes in habitat quality to determine conservation priorities^16–18^. It is the most suitable tool to meet our research goals and widely used in previous studies. For example, Sun et al.^19^ quantified the habitat quality of migratory birds, and assessed its spatial distribution characteristics under different land use scenarios in Poyang Lake wetlands based on InVEST model and Participatory Assessment framework method (Fo-PIA). Bai et al.^20^ analyzed the spatiotemporal characteristics of landscape patterns in Changchun city, and revealed the responses of habitat quality to urbanization based on InVEST model. Wu et al.^21^ used ArcGIS platform and InVEST model to simulate urban growth boundary of the Yangtze River Delta in 2024 and 2034 based on the evaluation of habitat quality. Additionally, Terrado et al.^22^ modified the InVEST model for the assessment of terrestrial habitat quality and extended it to freshwater habitats. These studies demonstrate the effectiveness of the InVEST model in assessing habitat quality. However, previous studies mainly focused on the model modification and extension, the influencing factors such as urbanization, land use change, and other aspects on the change of habitat quality and the impacts of habitat quality change on social-economic development. There is still room for research on patterns changes of urban habitat quality at long-term level, especially how to provide suitable protection objects and measures according to their change characteristics is urgent for the maintenance and sustainable management of urban habitat in metropolitan regions.

Shanghai is a metropolis with the highest urbanization level in China, and distributed with diverse habitat types, but its biodiversity is under great pressure^23^. Approximate 320,000 hm^2^ of inland wetlands and coastal tidal flats provide important habitats for wildlife and various ecosystem services for the city^24^. However, the built-up areas in Shanghai have rapidly sprawled in past decades, and numerous natural habitats presented a fragmentation trend and resulted in a continuous decline in the species and amounts of wildlife^25,26^. Shanghai is also experiencing an occasion when the numbers of native plants have decreased while alien plants have increased^27,28^, and 93% of invasive plant were distributed in these habitats of high nutrient and frequent disturbance in recent years. The results of Gao et al.^29^ indicated that, the low-quality habitats in Minhang District were attributed to the destruction of native habitats for wildlife during urbanization, and the preference for exotic species in urban greening construction. In general, the habitat homogeneity in suburb and fragmentation in downtown area resulted in the reduction of biodiversity in Shanghai^23–28^. However, few studies have revealed the differences in habitat quality patterns and their conservation potential in Shanghai at long-term level, and the inadequate knowledge on dynamic changes of urban habitat quality has constrained the planning and implementation of ecological protection projects.

As such, the objective of this study is to demonstrate a new perspective of integrating habitat quality and habitat conservation potential into identifying habitat protection zones and measures for habitat conservation in the megacity. To achieve this, this paper used InVEST model and spatial analysis methods to identify the habitat quality pattern in Shanghai from 2000 to 2017, novelly access habitat protected index (HPI) to reflect the potential value for conservation, and propose distinguished preservation objects and measures based on the conservation potential for urban habitat. This study can provide a reference for the conservation zoning scheme of urban habitats and sustainable ecosystem management in Shanghai.

## Materials and methods

### Study area

**Figure 1:**
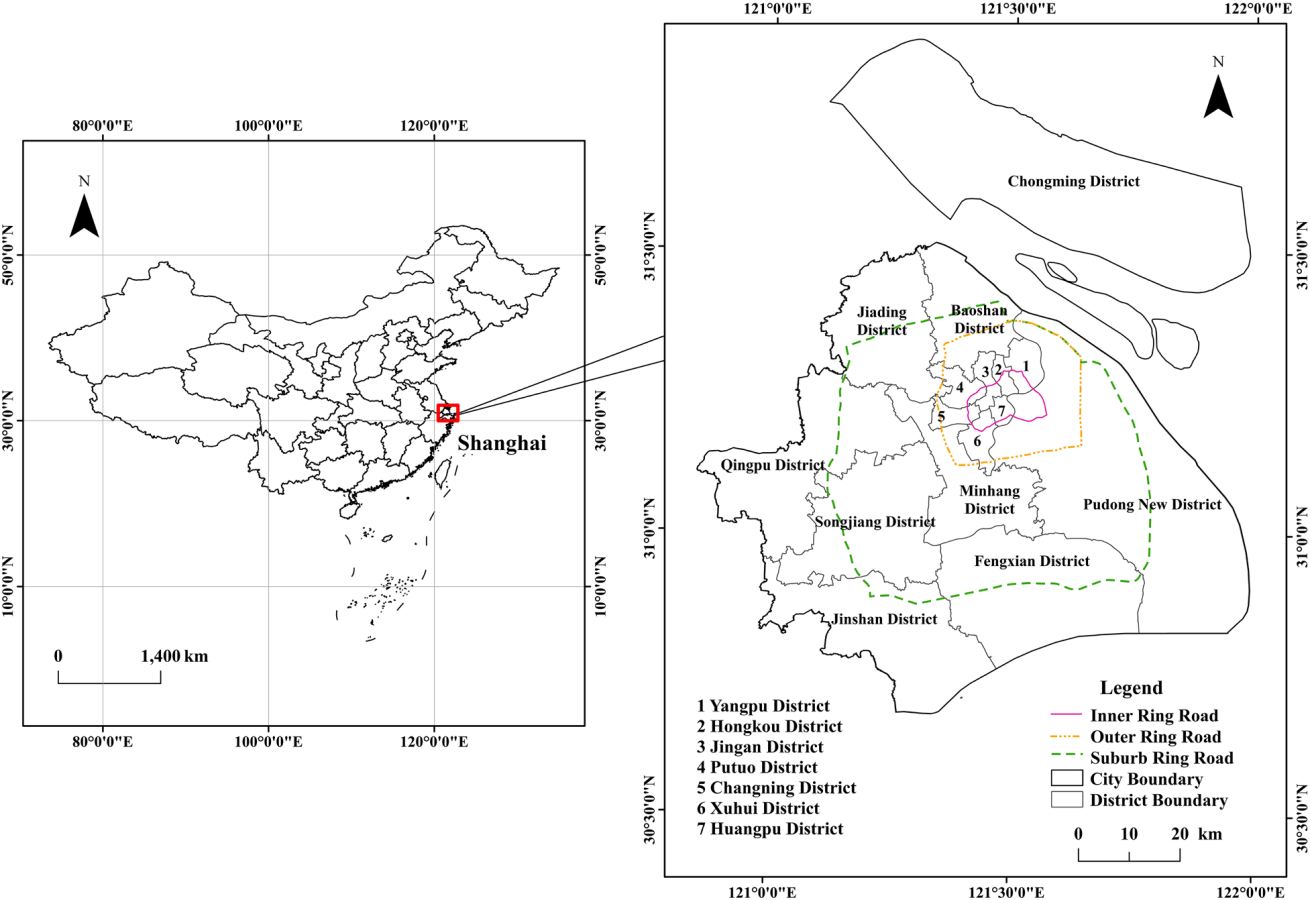
Map of study area in China (left) and administrative regions and the ring roads in Shanghai (right) created using ArcGIS version 10.8.1 (https://support.esri.com/en/products/desktop/arcgis-desktop/arcmap/10-8-1).

Shanghai is located in the eastern Yangtze River Delta (Fig. 1), and has the highest urbanization level in China. It encompasses approximately 6300 km^2^, and includes 16 districts. Except for a few remnant hills in the southwest, the whole region is broadly flat, with an average elevation of about 4 m. Shanghai belongs to the subtropical monsoon climate with a mean annual temperature of 17.7 ℃, sunshine time of 1809.2 h, mean annual precipitation of 1388.8 mm, and rainfall days of 124 days in 2017. Native vegetation is characterized by the subtropical evergreen broadleaved forest and the evergreen broadleaved-deciduous broadleaved mixed forest. In past decades, Shanghai has been devoting to the improvement of urban green landscape, the green coverage rate in urban built-up area increased from 12.4% in 1990 to 39.1% in 2017, and the public urban green space per capita increased from 0.7 m^2^ in 1990 to 8 m^2^ in 2017^30^. Shanghai is one of the largest cities in China in terms of population and economy. It had a total population of 24.15 million, a population density of 3814 people per kilometer, and a total GDP of 3000 billion Yuan in 2017. The population densities of Huangpu, Hongkou, Yangpu and Putuo District were as high as 20,000 people per kilometer.

According to Shanghai Biodiversity Conservation Strategy and Action (2012–2030)^31^, there were more than 300 species of freshwater fish, 530 species of terrestrial vertebrates, and 780 species of wild vascular plants in Shanghai. However, Shanghai is also trapped with urbanization issues, such as highly condensed population, imbalance of ecological, living and production space, poor quality of urban environment and so on^32^. Therefore, it is crucial for Shanghai to strengthen the monitoring on environment and ecosystem pressure, improve the ecological connectivity in urban green land system, and enhance urban ecological security and key ecosystem services^33^.

### Habitat quality index

The InVEST model is an open-source software model, and was developed by Stanford University, Minnesota University, the World Wide Fund and the Nature Conservancy^29^. Habitat quality is one of the modules of the InVEST model, and can represent the response level of different habitats corresponding to threat sources and the interaction of threat sources. It has been widely applied in assessments on ecological security, habitat quality and conservation effect of ecological projects at city and regional scales^34^. So we adapted the Habitat Quality module of InVEST model to map and access the habitat quality in Shanghai. The habitat quality is calculated as follows

1 $$Q_{xj} = H_{j} \times \left( {1 - \left( {\frac{{D_{xj}^{z} }}{{D_{xj}^{z} + k^{z} }}} \right)} \right)$$

where *Q*_*xj*_ is the habitat quality index (HQI) of land type *j* in grid cell* x*, *H*_*j*_ represents the habitat suitability of land type *j*, *z* and *k* are constants with a value of 2.5 and 0.5, respectively, *D*_*xj*_ denotes the threat level in grid cell* x* with land type* j*, and the equation is shown in Eq. (2):

2 $$D_{xj} = \sum\limits_{r = 1}^{R} {\sum\limits_{y = 1}^{{Y_{r} }} {\left( {\frac{{w_{r} }}{{\sum\limits_{r = 1}^{R} {w_{r} } }}} \right)r_{y} i_{rxy} \beta_{x} S_{jr} } }$$

where *R* denotes the number of threat factors, *y* indexes all grid cells on r’s raster map, *Y*_*r*_ indicates the set of grid cells on r’s raster map,* w*_*r*_ is the weight of threat *r* (Table l), *r*_*y*_ is the intensity of threat factor, *β*_*x*_ represents local factor of ecological protection in grid cell *x*, *S*_*jr*_ is the sensitivity of land type* j* to threat factor *r* where values closer to 1 indicate greater sensitivity (Table 2). Besides, *i*_*rxy*_ means the degradation decay function through distance, which can be expressed as the linear function of distance from threats to habitats, shown in Eq. (3):

3 $$i_{rxy} = 1 - \frac{{d_{xy} }}{{d_{r\max } }}$$

where *d*_*xy*_ is the linear distance between grid cells *x* and *y*, *d*_*r max*_ is the maximum impact distance of threat of *r* (Table 1).

**Table 1:** Threat factors, weight and their maximum distance of influence.

Threat factor	*d*_*r_max*_ (km)	Weight *w*_*r*_	Distance-decay function
Paddy fields	8	0.7	Linear
Dry land	8	0.6	Linear
Urban land	10	1	Linear
Rural residential land	5	0.6	Linear
Industrial and traffic land	6	0.5	Linear
Bare land	1	0.5	Linear

Identifying threats to habitats is a key issue in the InVEST model. According to the actual local situation and some other studies^35^, threat factors, weight of threat factors and maximum effective distance of threat factors were determined (Table 1). To identify the involved habitats, we selected land cover type as a habitat with consideration for overall biodiversity in the study area. The sensitivity score of each habitat to threats and the habitat suitability score were based on the InVEST user guide and a previous study^35^ (Table 2). However, previous studies often ignored the influence of local protection policies (*β*_*x*_)^5^. Considering that there are a large area of tidal flats, Chongming Dongtan Birds National Nature Reserve, and urban green parks such as Qingxi Country Park and Pujiang Country Park in Shanghai, we further utilized the local impact factor of ecological protection to optimize the assessment method. The value of *β*_*x*_ in nature reserves was set at l.5, and the value of *β*_*x*_ for parks, Huangpu River, and coastal area was set at 1.

**Table 2:** Sensitivity of land type to habitat threat factors.

Land types	Habitat suitability	Paddy fields	Dry land	Urban land	Rural resident land	Industrial and traffic land	Bare land
Paddy field	0.6	0	0.1	0.5	0.3	0.2	0
Dry land	0.4	0.2	0	0.5	0.2	0.2	0.1
Thick woodland	1	0.8	0.8	1	0.8	0.6	0.4
Shrubbery land	1	0.4	0.4	0.6	0.4	0.2	0.2
Sparse woodland	1	0.8	0.8	1	0.9	0.7	0.4
Grassland	0.7	0.4	0.4	0.6	0.5	0.3	0.2
Canal	1	0.6	0.5	0.8	0.6	0.4	0.2
Lake	1	0.6	0.5	0.9	0.7	0.5	0.3
Reservoir or pond	1	0.7	0.6	0.9	0.8	0.6	0.4
Shoaly land	1	0.8	0.7	1	0.9	0.7	0.6
Urban land	0	0	0	0	0	0	0
Rural residential land	0	0	0	0	0	0	0
Industrial and traffic land	0	0	0	0	0	0	0
Bare land	0.2	0.2	0.2	0.4	0.3	0.3	0

### Habitat protected potential and zoning

We assumed that the conservation potential of habitat can be determined by the importance of each grid in HQI, that is, if the habitat index in x grid contributes higher habitat quality to the total habitat quality in the municipality, it have higher potential for conservation in habitat quality. So we proposed HPI to reflect the potential value for conservation, and the higher HPI indicates more potential for habitat conservation (Table 3). The equation for calculating HPI is as follows:

4 $$HPI_{x} = \frac{{HQI_{x} \times 100}}{THQI} = \frac{{HQI_{x} \times 100}}{{\sum\limits_{x = 1}^{n} {HQI_{x} } }}$$

where *HPI*_*x*_ denotes the potential index for habitat protection in grid *x*(%), *HQI*_*x*_ is the sum of habitat quality index on grid *x*, *THQI* represents the total value of habitat quality index in study area, and n is the total number of grids.

**Table 3:** The criteria for habitat quality classification and its potential for habitat protection.

Habitat quality level	Habitat quality index(*HQI*)	Description	Habitat protect index range (*HPI*)/%	Description
I	*HQI* ≤ 0.2	Low habitat quality, obvious habitat deterioration, and human activities have a significantly negative impact on habitat	*HPI* ≤ 20	Large number of low-quality habitats, low importance and potential for habitat protection
II	0.2 < *HQI* ≤ 0.4	Lower habitat quality, visible habitat deterioration, and human activities have a negative impact on habitat	20 < *HPI* ≤ 40	Relatively large number of low-quality habitats, relatively low importance and potential for habitat protection
III	0.4 < *HQI* ≤ 0.6	Medium habitat quality, and its habitat is degraded and disturbed by human activities	40 < *HPI* ≤ 60	Certain number of high-quality habitats, medium importance and potential for habitat protection
IV	0.6 < *HQI* ≤ 0.8	Higher habitat quality, and its habitat has a tendency to degrade and is less disturbed by human activities	60 < *HPI* ≤ 80	Relatively large number of high-quality habitats, relatively high importance and potential for habitat protection
V	0.8 < *HQI* ≤ 1.0	High habitat quality, inapparent habitat degradation, and it can provide the best ecosystem service for human beings	80 < *HPI* ≤ 100	Large number of high-quality habitats, high importance and potential for habitat protection

To access the spatiotemporal differences of habitat quality and habitat protection potential in Shanghai, we divided the HQI and HPI into five levels shown in Table 3 according to their equidistant distribution, as well as previously reported study results and the actual situation of the study area^36,37^. 0 is the poorest habitat quality or HPI as well as 1 represents the highest quality or HPI.

Habitat quality protected areas must effectively contribute to sustaining biodiversity, ranging from preventing species extinction to retaining the most intact ecosystems. The conservation importance of regional habitat mainly depends on the current habitat types and habitat quality variations. For example, the native habitat and rapid shrink habitat should be given priority for conservation and restoration. Therefore, we generated the zoning map in habitat protected areas by integrating the habitat quality in 2000 and 2017, as well as the variation in HQI between 2000 and 2017. The evaluation processes were: (1) extracting 20% and 40% of the study areas with the largest HQI values and dividing the areas into three grades based on relevant studies about assessing Ecological Conservation Redline and the actual situation in Shanghai^38,39^. (2) Grading habitat quality variations from 2000 to 2017 by a criterion of [− 1 to 0.2), [0.2–0.8), and [0.8–1) considering the effectiveness of the ecological space construction and optimization projects in Shanghai between 2000 and 2017; (3) adding up all three index values in ArcGIS 10.8 and manually grade the total index value with consideration of their relative importance to maximize the representativeness and effectiveness of habitat conservation; (4) removing the fragmented patches with an area less than 10 hm^2^, as fragmented habitats with an area less than 10 hm^2^ are easier to influenced and filled by built-up land^40^.

### Data and validation

Land cover datasets for the years 2000, 2005, 2010 and 2015 were obtained from the land-use remote sensing monitoring database (1:100,000 scale). The land cover in 2017 has been generated from Gaofen-2 satellite images with 2-m resolution captured from 29th April 2017 to 10th March 2018 provided by Land Observation Satellite Data Platform of China Center for Resource Satellite Data and Applications. Firstly, ortho-calibration, radiometric calibration, image fusion and atmospheric correction processes were conducted on ENVI to generate high-resolution imagery after registration. Then, the supervised classification method with maximum likelihood clustering and digital elevation model data was used as a hybrid method to classify images. Pure pixels are selected as the training sample instead of mixed pixels. Mixed classes, such as forest and grass, are separated by manual visual interpretation. According to CNLUCC Classification System and the actual situation in Shanghai, the land cover was divided into 6 first-level classes and 14 s-level classes (Table 4). Urban land, Rural residential land, Industrial and traffic land, Paddy field, Dry land and Bare land were treated as threat factors. The accuracy of land cover interpretation was verified by using the stratified and classified random sampling method. Based on field survey data and Google Earth images, we used the Random module in ArcGIS 10.8 to calculate the number of samples for each land type and randomly create samples. A total of 6365 valid sample points were selected for the accuracy test and the interpreting accuracy of each primary land-use class was more than 82%, which showed relatively high accuracy. Finally, those classification data were converted to threat factor rasters with a grid of 30 m × 30 m with the help of ArcGIS 10.8.

**Table 4:** Classification of land cover in Shanghai.

First-class land cover	Second-class land cover	First-class land cover	Second-class land cover
Agriculture land	Paddy field	Wetland	Canal
	Dry land		Lake
Woodland	Thick woodland		Reservoir or pond
	Shrubbery land		Shoaly land
	Sparse woodland	Building land	Urban land
Grassland	Grassland		Rural residential land
Bare land	Bare land		Industrial and traffic land

**Figure 2:**
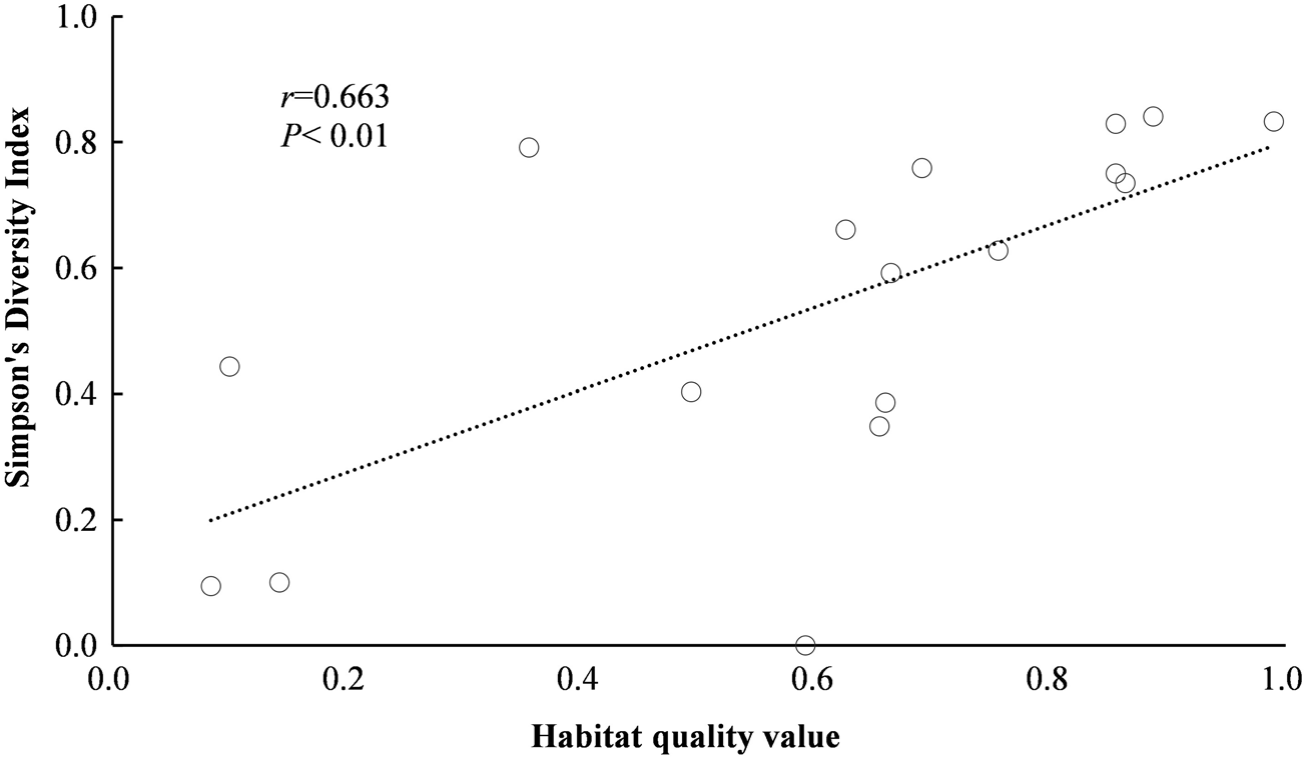
Relationship between habitat quality and Simpson's Diversity Index.

In addition, we validated the evaluation results of habitat quality through a biodiversity survey. Generally, areas with high-quality habitats were described as high biodiversity^8^. In August 2019, we carried out a quadrat community survey in 20 typical ecological spaces in Shanghai^32,41^. Three 30 m × 30 m plots were randomly selected in each quadrat, and vegetation community information such as species and quantity of trees and shrubs, vegetation coverage and so on were recorded. Considering the highly matured urbanization level in Shanghai, barely the land use of survey plots conversed from natural ecosystem to built-up area in latest years. Considering the relationship between biodiversity and habitat quality, Simpson's Diversity Index was calculated for each plot to test the accuracy of habitat quality. The results showed that the Pearson correlation coefficient between Simpson's Diversity Index and habitat quality in 2017 was 0.663 (P < 0.01) (Fig. 2), which indicated that the precision of the evaluation results was credible.

## Results

### General variations in habitat quality

**Figure 3:**
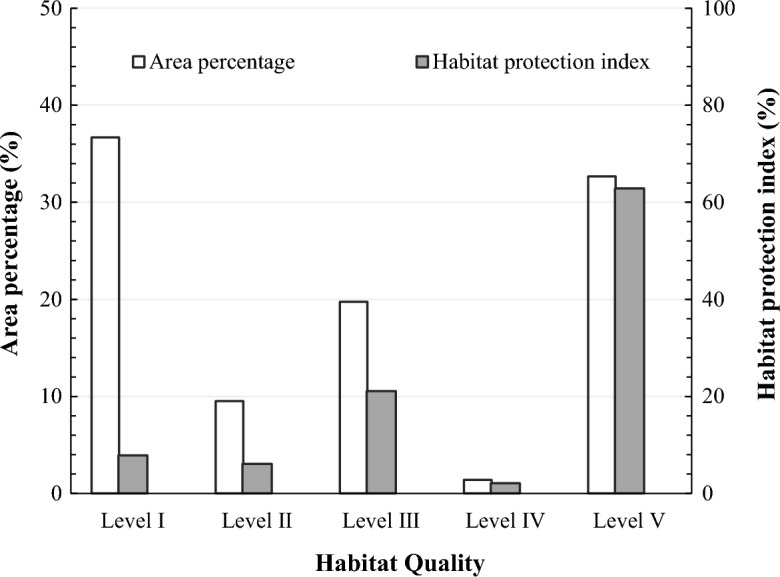
Distribution of habitat quality classification in Shanghai.

In order to analyze the variation trend of habitat quality in 2017, we calculated the HQI and HPI at different levels (Fig. 3). Our results indicated that, the average HQI of Shanghai was 0.42 in 2017, and belonged to level III, which indicated that the biodiversity resources in Shanghai were highly threatened. We also found that the areas of level I and II occupied 36.68% and 32.66% of the total area, respectively, and they were the major habitat quality types in Shanghai. The area of habitat quality level I was widely distributed in the middle of Shanghai, with an area of 2862.92 km^2^, yet the regions with habitat quality level I presented the lowest HPI (7.84%). The area of habitat quality level V covered approximately 2496.58 km^2^, and was mainly distributed in the eastern and southern edges, and regions with dense rivers and lakes across Shanghai. Meanwhile, the area of habitat quality level V had the highest HPI (62.84%). The areas of habitat quality levels II and III, which were mainly distributed in the outer suburb of Shanghai and Chongming Island, reached 741.83 km^2^ and 1540.97 km^2^, respectively, and their HPI were 6.10% and 21.11%, respectively. The area of habitat quality IV sporadic distributed, occupied a minor part of Shanghai with a proportion of 1.41%, and generated the lowest HPI. On the whole, the habitat quality in Shanghai was at a moderate to low level. The area proportion of the lowest and highest habitat quality levels were both relatively large, exhibiting obvious polarization in the urban habitat quality. Therefore, it is urgent to strengthen the conservation of high-quality habitats in the urban fringe and Chongming district.

### Spatial differences in habitat quality

**Figure 4:**
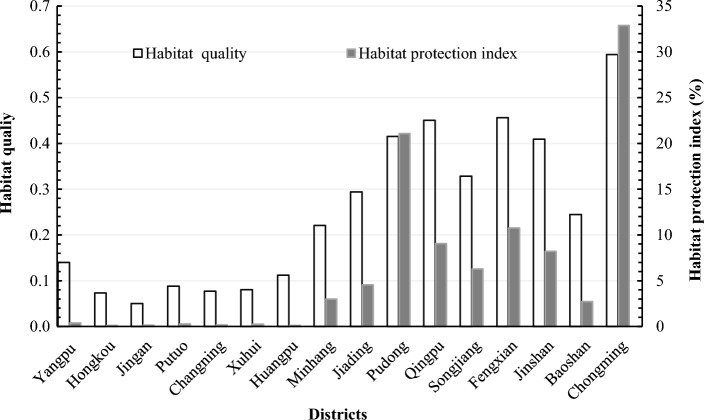
Area percentages of habitat quality among administrative regions in Shanghai.

To identify the spatial distribution of habitat quality in different zones of Shanghai, we carried out a statistical analysis of the HQI among different administrative regions and traffic loops. The habitat quality of Shanghai had significant regional differences. HQI of Yangpu, Hongkou, Jingan and Putuo, Changning, Xuhui and Huangpu district in downtown areas were less than 0.2, especially habitat quality in Jingan district was the lowest (HQI = 0.05). Natural habitats in the core area were highly threatened by outside disturbance, so the potential value of habitat protection in this area was low. The HPI of Hongkou district was the lowest, with a value of 0.11%. HQI and HPI of Minhang, Baoshan, Jiading and Songjiang district were at level II and 3–6%, respectively. It indicated that habitats in these areas were greatly disturbed by human activities, and their potential values of habitat conservation were correspondingly low. HQI of Jinshan, Qingpu, Fengxian and Pudong New district were between 0.4 and 0.5, which was at the moderate level, whereas the HPI in Pudong New district was relatively high (21.08%). In addition, the HQI of Chongming district was close to 0.6, which was the highest habitat quality of all districts, and the HPI in Chongming district was accordingly as high as 32.90%. Thus, Chongming district was the main contributor to habitat quality and high-conservation habitats in Shanghai (Fig. 4).

Chongming district is primarily composed of Chongming, Changxing and Hengsha islands, and the land cover here was mostly forest, farmland and wetland, which were relatively less disturbed by urban development. Hence, Chongming district had relatively independent attributes compared with the metropolitan area. Accordingly, it’s essential to analyze the spatial distribution of habitat quality in metropolitan area that excludes Chongming District. Table 5 shows that HQI increased significantly from areas inside Inner Ring Road to areas outside Outer Ring Road. HQI in areas inside Inner Ring Road and areas between Inner Ring Road and Outer Ring Road were 0.07 and 0.15, respectively, revealing that the habitat quality of areas inside Outer Ring Road was low and mainly level I. Secondly, the HQI in area between Outer Ring Road and Suburb Ring Road was relatively low and mostly levels I and III, yet the HPI was relatively high and the value reached 34.21%. The habitat quality in areas outside Suburb Ring Road was at moderate grade, whereas the area proportion of habitat quality levels V increased significantly and its HPI was the highest.

**Table 5:** Habitat quality index differences among traffic loops in center area of Shanghai.

Areas divided by ring road	Averaged HQI	Habitat quality level and its area percentage (%)	HPI (%)
Areas inside the inner ring road	0.07	I (90.87)	0.79
Areas between inner and outer ring road	0.15	I (82.04)	5.26
Areas between Outer ring road and suburb ring road	0.30	I (52.78); III (21.31)	34.21
Areas outside suburb ring road	0.47	I (29.27); III (21.73); V (35.46)	59.74

### Temporal changes of habitat quality

**Figure 5:**
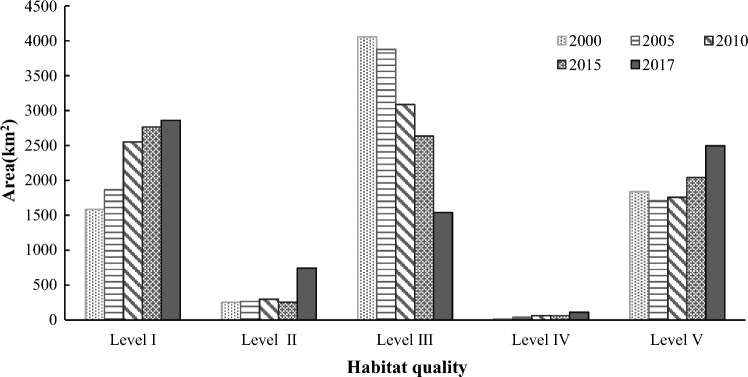
Annual changes of habitat quality in Shanghai from 2000 to 2017.

**Figure 6:**
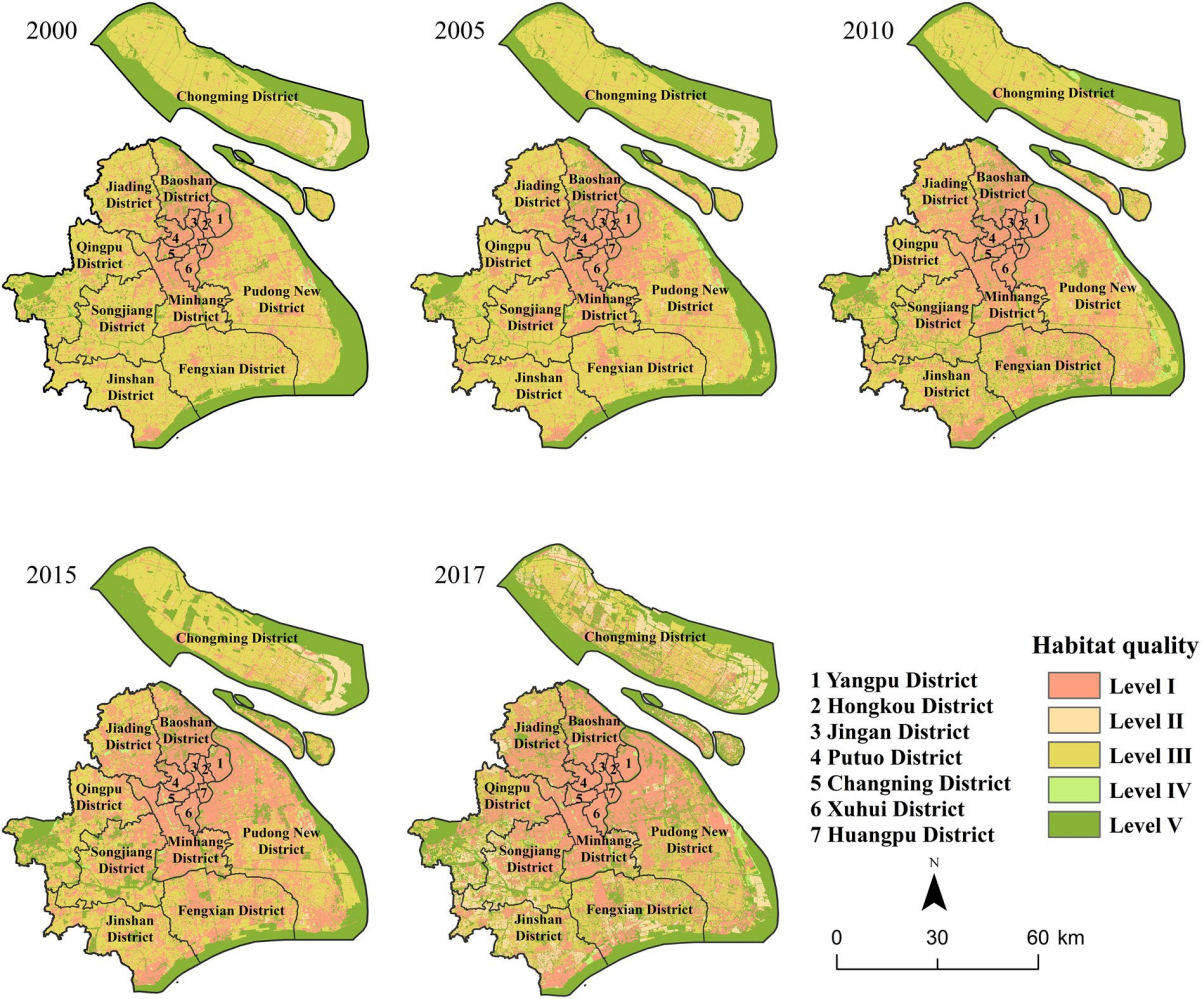
Spatial distribution of habitat quality in Shanghai from 2000 to 2017 created using ArcGIS version 10.8.1 (https://support.esri.com/en/products/desktop/arcgis-desktop/arcmap/10-8-1) and InVEST version 3.10.1 (http://releases.naturalcapitalproject.org/?prefix=invest/3.10.1.post25+g08a03605a/).

Based on InVEST Habitat Quality model and ArcGIS spatial statistical analysis, significant spatial and temporal differences of habitat quality in Shanghai have been found. In general, the HQI of Shanghai decreased from 0.56 in 2000 to 0.42 in 2017, with an annual average value of 0.49. It suggested that the natural habitats were constantly occupied by the construction land in Shanghai in past decades. From 2000 to 2017, the area proportion of habitat quality level I in Shanghai increased from 20.43 to 36.93%, and the area proportion of habitat quality level II increased from 3.28 to 9.57% (Fig. 5). These results indicated that the scale of low-quality habitats increased significantly, and expanded from downtown area towards suburban area (Fig. 6). During the study period, the area proportions of habitat quality levels IV and V increased correspondingly, and the increased areas were mainly concentrated in the eastern and southern areas of the urban fringe. It suggested that the ecological protection measures in the suburbs and coastal areas of Shanghai have achieved some effectiveness. However, the area proportion of habitat quality level III declined from 52.31% in 2000 to 19.88% in 2017, which was mainly due to the large number of suburban cultivated land being transformed into industrial land and urban residential land.

**Figure 7:**
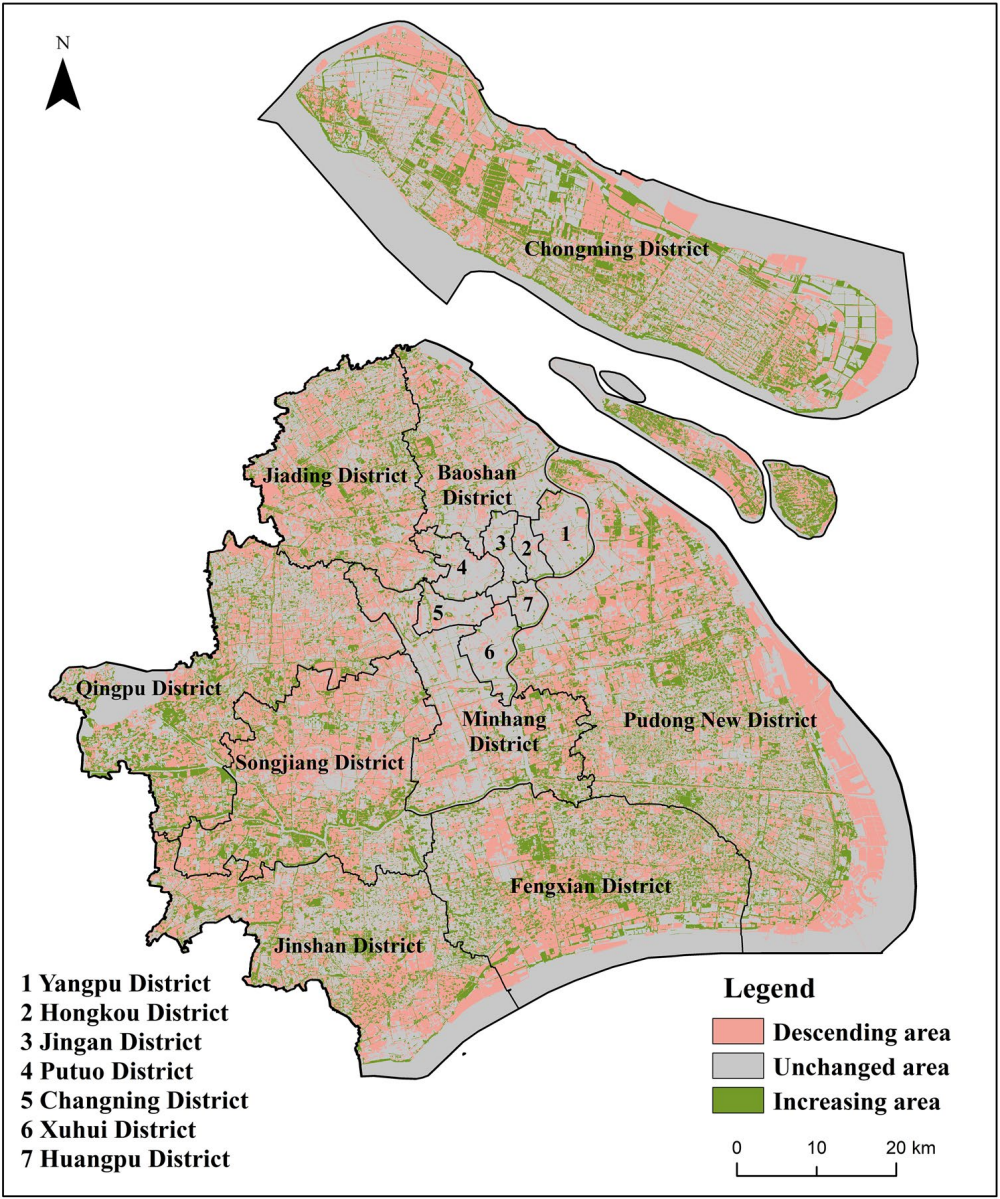
Spatial differences in habitat quality changes in Shanghai during 2000–2017 created using ArcGIS version 10.8.1 (https://support.esri.com/en/products/desktop/arcgis-desktop/arcmap/10-8-1) and InVEST version 3.10.1 (http://releases.naturalcapitalproject.org/?prefix=invest/3.10.1.post25+g08a03605a/).

Figure 7 exhibits the spatial differences of habitat quality between 2000 and 2017. Compared with habitat quality in 2000, approximately 33.l2% of the total area in Shanghai experienced a decline in habitat quality in 20l7. The descending areas were mainly distributed in the west and south suburbs of Shanghai, coastal area and north area of Chongming Island, which all were rapid urbanization areas of Shanghai in recent years. About 19.21% of the total areas improved in habitat quality, and the improved areas were mainly distributed in distant suburbs, such as Fengxian District, Qingpu District, Pudong New District, the central parts of Chongming Island and Hengsha Island. The remaining 47.67% of the total area, mainly distributing in the coastal area, reservoir and downtown areas, was not changed significantly in habitat quality. The reason for this was that downtown area had been largely built up by 2000, and had few large-scale land use adjustments in recent years, whereas the coastal area and reservoir have been subjected to strict ecological protection.

### Zoning schemes for habitat conservation

**Figure 8:**
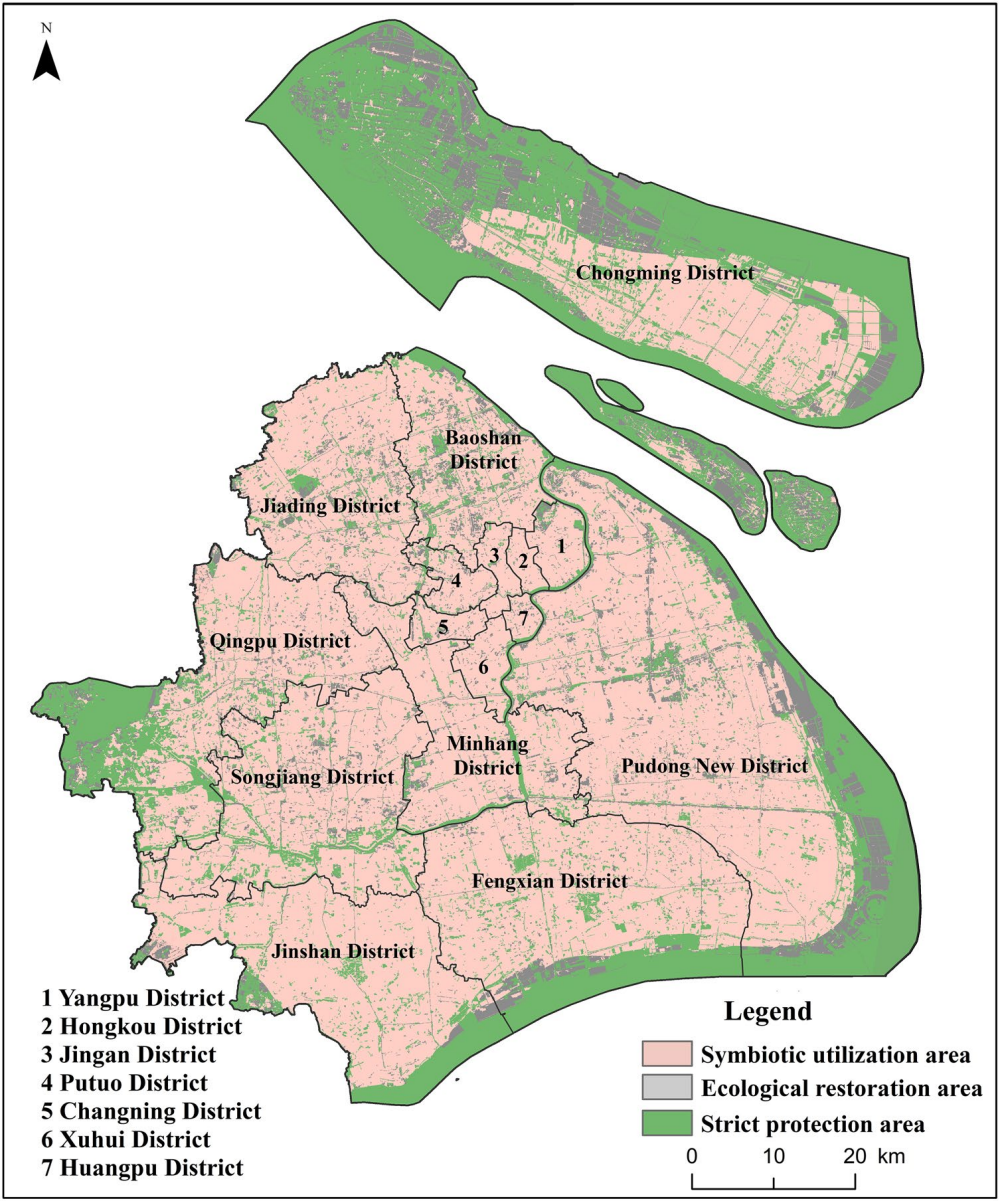
Habitat quality protected area in Shanghai created using ArcGIS version 10.8.1 (https://support.esri.com/en/products/desktop/arcgis-desktop/arcmap/10-8-1) and InVEST version 3.10.1 (http://releases.naturalcapitalproject.org/?prefix=invest/3.10.1.post25+g08a03605a/).

Considering the immense differences in habitat quality, threatened degree and urgency of conservation in different regions, it is necessary to implement zonal ecological conservation and utilization measures. Figure 8 illustrates habitat protected areas according to the habitat quality in 2000 and 2017, as well as the variation in HQI between 2000 and 2017.

The area in need of strictly ecological protection (strict protection area), mainly distributing in eastern and southern coastal wetlands, Dianshan lake, Huangpu River upstream region and Chongming district, covered 2173 km^2^, accounting for about 30% of the metropolitan area. Furthermore, this area was characterized by the majority of high-quality habitat and the strategic blank area of ecological conservation in Shanghai with an HPI of 41–56%. As a result, this region is suitable for implementing the strictest ecological environmental protection and management, strictly controlling human development and construction activities, minimizing anthropogenic disturbance, and strengthening long-term monitoring of ecosystem and biodiversity. Shanghai is one of the most developed metropolis cities in China, so most of the study area was a symbiotic utilization area mainly for human habitation and supplemented by biodiversity protection. The corresponding land type of the symbiotic utilization area was construction land and cultivated land. This area occupied 53% of the total area with an HPI of 21–37%, and the key measures in this area should focus on optimizing the spatial layout of urban blue space (such as rivers and lakes) and green space (such as parks), strengthening the ecological management of urban parks and rivers, minimizing disturbance from human activities and leaving a certain amount of space for urban wildlife to achieve the harmony of human and nature in Shanghai.

The area except for the strict protection area and symbiotic utilization area was the ecological restoration area. The ecological restoration area approximately covered 1471 km^2^, accounting for 17% of the total area. It was mainly distributed in the east and south of the inner coastal zone, the north and southeast of Chongming Island, and the corresponding land type was farmland, wetland, forest and grassland. However, HPI in this region was relatively low (23%) and the habitat quality showed a dramatic decline trend between 2000 and 2017. Therefore, it is urgent to strengthen the monitoring on habitat degradation, implement ecological restoration projects in key habitat areas, and enhance the ecological buffer function between the symbiotic area and the strict protection area.

## Discussion

With the promotion of urbanization and ecological civilization construction, natural habitats and biodiversity in rapid urbanization areas are receiving increasing attention^3^. In this study, we investigated the habitat quality in Shanghai by using InVEST model. Results indicated that the habitat quality in Shanghai varied between 0.42 and 0.56 from 2000 to 2017, and the habitat quality in 2005 and 2015 was higher than the conclusion of Ou et al.^35^, which was probably due to the larger sea area in this study area. The HQI of Shanghai in 2010 was lower than 0.58 obtained from Wu et al.^21^, which mainly because the cultivated land was regarded as a threat source in this study.

This study concluded that HQI in Jinshan, Qingpu, Fengxian and Pudong New district were at medium level, while the majority of high-quality habitat and high protected habitat aggregated in Chongming district. According to the investigation by Li et al.^27^, key wild plants under protection in Shanghai were mainly distributed in Jinshan Island, Sheshan area, Sheshan Island and other areas with high biodiversity. Due to the obvious expansion trend of impervious surface in the metropolitan area of Shanghai^42^, HQI gradually increased from Inner Ring Road to Outer Ring Road, which was consistent with the conclusion that aphid Shannon–Wiener index was the highest in the suburban area and the lowest in the central area^43^. However, on account of habitat loss and habitat degradation caused by urbanization, especially forest or wetland reduction and fragmentation, the habitat quality of suburban areas in the west and south of Shanghai, the coastal area, and the north of Chongming Island have declined rapidly since 2000^3^.

Habitat quality assessment plays a crucial role in the study of biodiversity and its conservation^9^. Although a large number of existing studies have revealed the influences of urban expansion on natural habitats, how to conserve high-quality habitat in the context of rapid urbanization is an important question^44^. In order to promote the environmental quality in Shanghai, ensure the integrity and stability of ecosystem and curb the declining trend of habitat quality, this paper proposed distinguished conservation objects and measures in different zones based on the habitat quality patterns and its spatiotemporal variations between 2000 and 2017. The ecological restoration area characterized by damaged habitats needs to be preserved and restored by means of ecological engineering. For symbiotic utilization area, optimizing the spatial layout of urban blue and green spaces should be emphasized. For strict protection area with high-quality habitats, destructive human activities should be constrained, and the original single conservation mode should be broken through establishing a natural habitat protection system suitable for urbanized areas. The strict protection areas and zonal protection schemes were generally consistent with the identification of regional biodiversity hotspots and biodiversity conservation measures reported by other studies^38,45–48^.

We also admitted to the limitation of data in this study, which derived from the remote sensing data ranging from 2000 to 2017. However, the urbanization rate in Shanghai has reached 89% since 2010, the highly matured urbanization level resulted in relatively slow land change. So the future study should use the remote sensing in latest year to identify more specific habitat conservation zones for ecological protection and sustainable governance. Furthermore, It must be addressed that the InVEST model still has some limitations in habitat quality assessment^11,49^. Firstly, the model indicators in this study were set with references to the model guidance manual and similar literature, which gives a subjective tendency in the study. Further empirical research on more precise indicators is needed to improve the accuracy of habitat quality assessment. Secondly, the spatial resolution of imagery affects the accuracy of land cover classification and vegetation coverage. This study selected 30 m × 30 m resolution data to derive habitat quality, so the scale-effect of remote sensing imagery in spatial dimension should be attached more importance in future research. Thirdly, various elements aggregation in urbanization often results in regional variations in habitat quality, especially clusters of socio-economic factors that have a significant impact on the spatial heterogeneity of habitat quality^18^. This study mainly adapted land cover to reflect human activity, so the future study should consider inputting more representative socio-economic factors for complex systematic simulation in further model improvements and optimization.

## Conclusion

The results showed that the habitat quality in Shanghai was at moderate level, and the HQI presented the lowest or highest levels in most regions, but the areas of medium habitat quality were relative inadequacy. Chongming district had the highest HQI and HPI in Shanghai. From the perspective of long-term scales, the habitat quality in Shanghai showed an obvious decline tendency. The area of medium level of habitat quality decreased significantly, whereas the area proportion of the lowest and highest levels of habitat quality increased significantly. Results revealed both anthropogenic disturbance and ecological conservation effects continued to amplify. In order to efficiently preserve habitat resources in Shanghai, nearly 30% of the total area needs strict ecological protection, which were mainly distributed in the eastern and southern coastal wetlands, Dianshan Lake, the upper stream of the Huangpu River and Chongming District, and about l7% of the total area, mainly distributed in the inner coastal area, should be implemented ecological restoration and sustainable governance.

## Data Availability

The datasets used and/or analyzed during the current study are available from the corresponding author on reasonable request.
